# The Therapeutic Relationship in China: A Systematic Review and Meta-Analysis

**DOI:** 10.3390/ijerph18073460

**Published:** 2021-03-26

**Authors:** Ying Mao, Wei Ning, Ning Zhang, Tao Xie, Jinnan Liu, Yongbo Lu, Bin Zhu

**Affiliations:** 1 School of Public Policy and Administration, Xi’an Jiaotong University, Xi’an 710049, China; ningwei@stu.xjtu.edu.cn (W.N.); ningzhang.xjtu@foxmail.com (N.Z.); xietao2014077049@stu.xjtu.edu.cn (T.X.); kennanliu@stu.xjtu.edu.cn (J.L.); whl1692881375@stu.xjtu.edu.cn (Y.L.); 2 Research Center for the Belt and Road Health Policy and Health Technology Assessment, Xi’an Jiaotong University, Xi’an 710049, China; 3 School of Public Health and Emergency Management, Southern University of Science and Technology, Shenzhen 518055, China; binzhu2-c@my.cityu.edu.hk

**Keywords:** therapeutic relationship, medical disputes, determinants, systematic review, meta-analysis

## Abstract

With a surge of conflicts between healthcare workers and patients in recent years, the therapeutic relationship (TR) in China is presently in tension. Meanwhile, consequent issues have begun to emerge, such as the distrust between healthcare workers and patients and the decline in the quality of medical services. Although many empirical studies about the TR have been conducted in China, previous studies on TR and its influencing factors have been contradictory. Therefore, this study conducted a systematic review and meta-analysis to assess the current situation of the TR and to identify factors associated with the TR in Chinese hospitals from three perspectives (healthcare worker, patient, and therapeutic interaction). Two reviewers independently searched the literature, selected researches, and extracted data through comprehensively searching of three international electronic databases and three Chinese electronic databases to identify all relevant observational studies on influencing factors for TR in China published in English and Chinese from January 2000 to January 2020. Among the 3290 records initially identified, 11 studies met the selection criteria. A total of 96,906 individuals were included in the review. The results showed that 55.73% of healthcare workers consider the TR to be tense, and 33.7% of patients hold this view. The meta-analysis indicated that healthcare workers who were male, older, less educated, working in a non-surgical department, and had a senior title were more likely to be pessimistic about the TR. Patients who were rural residents, highly educated, and had no medical insurance were more likely to be pessimistic about the TR. Furthermore, the mutual trust could improve rapport between healthcare workers and patients. The 25 other related factors related to the TR were analyzed and described using a narrative approach. The findings might deserve consideration in the design of relative policies to promote harmony between doctors and patients.

## 1. Introduction

In China, contradictions between doctors and patients have dramatically increased nationwide in recent years. Patients sometimes use harmful behaviors against doctors, resulting in bad consequences, which has aroused the attention of the medical industry and various groups in China. Coordinated and harmonious therapeutic relationship (TR) is an important factor to improve the quality of medical treatment. However, the TR is quite tense in the Chinese context [1,2,3], and patients’ satisfaction with medical treatment and healthcare worker-patient trust is damaged [4,5]. Also, healthcare workers do not earn deserved respect, verbal attacks, emotional conflicts, injuries and even killings happen from time to time. Moreover, disharmonies and contradictions of TR will disrupt patients’ adherence to treatment and continuity of medical care [3,6], and further affecting the quality of health services. As a result, people’s sense of well-being and health has severely been affected.

Medical disputes, the primary but not the only symbol of the deteriorating doctor-patient relationship, have become increasingly prominent in recent years, impacting the development and optimizing of TR. Previous studies have reported that medical professionals are more vulnerable to workplace violence than other professionals [7,8]. In the United States, the number of medical disputes more than tripled from 1991 to 2005 [9]. A survey of workplace violence between nurses and patients in the United States showed that 76% of nurses had been attacked by patients or patient family members [10]. In 2017, 288 physical injuries and approximately 2600 psychological insults occurred in Germany, and nearly 40% of doctors experienced physical conflicts [11]. This rapid growth of medical disputes existed not only in Western countries but also in Asia. For example, in Japan, it was reported that the number of medical malpractice lawsuits had increased ten times from 102 in 1960 to 1019 in 2003 [12].

In the Chinese context, people have been largely dependent on the public services provided by the government since ancient times [13,14], and their demand for health services is no exception. Public hospitals funded by the government have always occupied a dominant position in the medical industry [15]. However, since the reform of China’s economic system in 1993, excessive marketization prompted the ambitions of medical personnel chasing interests [16,17], as well as led to the majority of patients flocking to tertiary hospitals. Therefore, the so-called “difficult and expensive to get medical treatment” problem has been developed throughout China [1,18,19]. The public benefit of public hospitals has gradually weakened, and the bitterness and contradictions between doctors and patients have deepened. Additionally, the institutionalized communication and mediation mechanism of public hospitals in China are generally not sound [1]. After medical disputes occur, patients lack channels to express their appeals, which contributes to the intensification of contradictions between healthcare workers and patients in China, and even the frequent occurrence of violent injuries to healthcare workers. A survey conducted by the Chinese Medical Doctor Association in 2017 reported that 66% of medical personnel had experienced verbal abuse or physical injury [20]. Moreover, the public ordinarily tends to be partial to the weak, and people show more sympathy and compassion to patients in medical disputes [17]. People generally show more empathy to patients’ situations before they fully understand what happened. With the various social grievances inevitably aggravated by the problem of “difficult and expensive to get medical treatment” in China, as well as the one-sided reports by the media [21], healthcare workers and patients, become two parties with opposing interests rather than a “community of interests.”

The TR also has strong sociocultural attributes in essence. Under the joint action of social transformation and healthcare system reform, the conflict and contradiction between doctors and patients were no longer the individual conflict between doctors and patients, but have risen gradually to a wide range of group areas and social problems [22]. On the one hand, in China’s current model of healthcare, the progress of modern medical science and technology has increased the medical staff’s dependence on medical equipment, resulting in that the therapeutic interaction has been seriously materialized. The integrity of people has been replaced by organs, tissues, cells, and genes, while the humanistic and psychological interaction between doctors and patients is relatively lacking. A national survey found that the average inquisition time for patients in tertiary hospitals was only 11.5 min in 2019, which is far less than the average waiting time for patients (24.1 min) [23]. The doctors tend to be concerned more with the disease in a biomedical sense, rather than the whole patient as a person [24]. Therefore, there are model-based differences [25] between China and countries implementing the person-centered care (PPC) that a model emphasizes that medical services should not only pay attention to the treatment of patients’ diseases, but also pay more attention to the patients’ living conditions, psychological needs, emotional feelings, and other mental states at a higher level. On the other hand, the high degree of medical information asymmetry and distrust of the medical process (caused by social cognition) result in that there are differences or even conflicts between doctors and patients on the spiritual interests such as status in power or right, human dignity, pursuit of equality, and realization of personal value. As a result, doctors are in the dominant side of the game spontaneously in the therapeutic interaction process, such as information advantage and position advantage. A research report, issued jointly by the World Bank (WB), the World Health Organization (WHO), and the authorities of the Chinese government, put forward to set the “People-Centered and Integrated Health Care” (PCIC) for the next stage of China’s healthcare system reform key strategy [26]. However, the implementation of the PCIC in China is still in its initial stage, and the Chinese healthcare setting is still widely affected by the old biomedical model.

Furthermore, this “contradiction pattern” may trigger a vicious cycle [27] in that poor TR would undermine the mutual trust between healthcare workers and patients, which would, in turn, result in medical disputes. Therefore, it is important to analyze the determinants of the TR and to establish effective strategies to improve such relationships. Previous studies have explored the effects of various factors on the TR, such as healthcare worker-related factors (i.e., age [28,29,30,31,32,33,34], gender [34,35,36,37,38,39,40], years of experience [41,42], education level [43,44,45], ethnicity [45], marital status [44], professional title [46,47], department [28,48,49], income [45,50,51], income satisfaction [45,52,53], etc.,), patient-related factors (i.e., age [44,45,51], gender [44,45,51], residence [54,55,56], ethnicity [45], education level [57,58], medical insurance [47,59], medical expense [59], household income [45], waiting time [45], etc.,), and other factors (i.e., doctor-patient trust [60,61], doctor-patient communication [62,63], etc.,). Obviously, previous studies have been conducted to investigate the individual factors and socioeconomic factors for both doctors and patients, as well as factors related to the therapeutic interaction.

Although many empirical studies have been conducted in China, most were published in Chinese journals, and no systematic reviews related to the TR have been found. Therefore, this study aimed to conduct a systematic review and meta-analysis to assess the current situation of the TR and its determinants (from three perspectives of healthcare worker, patient and healthcare-patient interaction) and to identify factors associated with the TR in Chinese hospitals.

## 2. Materials and Methods

According to the Preferred Reporting Items for Systematic Reviews and Meta-Analyses guidelines we following (Known as PRISMA, Ottawa Hospital Research Institute) [64], this study comprehensively searched published studies that investigated the determinants of the TR in electronic databases. This systematic review was conducted based on English and Chinese databases from 1 January 2000 to 1 January 2020. We searched the following English databases: PubMed, EMBASE and Web of Science; we also searched the following Chinese databases: China National Knowledge Internet Database (CNKI), Wanfang Database, and China Biology Medicine disc (CBM/Sinomed).

### 2.1. Search Strategy

The same keywords were used for each database search for China “[(China) OR (Chinese)],” therapeutic relationship “[(doctor-patient relationship) OR (clinician-patient relationship) OR (therapist-patient relationship) OR (physician-patient relationship) OR (doctor-patient relation) OR (clinician-patient relation) OR (therapist-patient relation) OR (physician-patient relation) OR (the relationship between doctor and patient) OR (the relationship between physician and patient) OR (the relation between doctor and patient) OR (the relation between physician and patient) OR (the relation between clinician and patient) OR (the interactions between doctor and patient) OR (the interactions between clinician and patient) OR (the interactions between physician and patient) OR (medical dispute) OR (conflict between doctor and patient) OR (conflict between physician and patient) OR (conflict between clinician and patient) OR (medical trouble)],” and determinant “[(determinant) OR (factors) OR (influences)]”. The specific search strategy is shown in the Supplementary File.

### 2.2. Eligibility Criteria

According to the objective of this study, the characteristics of selection criteria were determined through discussions, which were summarized in Table 1. The eligibility criteria were as follows: (1) studies (without language restrictions) that were published in a peer-reviewed journal from 1 January 2000 to 1 January 2020 and located in China were included. (2) Studies in which participants included doctors, nurses (excluding doctors or nurses in internship and medical students), and patients were included. (3) Studies that analyzed the current situation of the TR and its related factors affecting the TR were included. (4) Studies with original data were included. (5) Only cross-sectional studies were included.

### 2.3. Data Extraction and Quality Assessment

Two independent reviewers (Wei Ning and Ning Zhang) participated in the data extraction by screening the acquired studies at the same time, according to the flow diagram. All discrepancies were resolved through discussions, leading to full group consensus. All articles were extracted into an extraction form focused on identifying the following for each study: the first author, year of publication, locations, participants, sample size, qualified rate, analytical perspective, determinants, negative cases, rate of negative cases, and the number of references.

According to the Grading of Recommendations: Assessment, Development, and Evaluation (GRADE) approach [65], a modified Newcastle-Ottawa scale (NOS) was utilized to assess the quality of the included studies. The quality assessment criteria consisted of seven components: (1) representativeness of the sample, (2) sample size, (3) non-respondent rate, (4) ascertainment of the exposure, (5) comparability of subjects in different outcome groups, (6) assessment of the outcome, and (7) use of an appropriate statistical test. The total quality assessment score of each study was 7, which was divided into three levels: good quality (score of 5–7), medium quality (score of 3–4), and poor quality (score of 1–2). Studies with medium and good quality were included in our analysis.

### 2.4. Statistical Analysis

We used RevMan 5.3 (The Cochrane Collaboration, Oxford, UK) and Stata 16.0 (Stata Corp, College Station, TX, USA) to statistically analyze the results from the included studies. This study mainly analyzed the determinants of the TR from the perspectives of healthcare workers, patients, and therapeutic interactions. All related variables extracted from the included articles were added to the standard extraction form, and the different variables were transformed into binary variables to permit dichotomous meta-analysis. However, only the same variables or variables that could be combined into the same types were included in the meta-analysis. The variable screening process is shown in the Supplementary File.

The determinants included in the meta-analysis were those reported in at least three articles. For the meta-analysis, we conducted a dichotomous meta-analysis and computed the summary risk estimate by using a fixed-effect model. The significance of the pooled odds ratio (OR) was determined by the Z-test, with *p* < 0.05 considered statistically significant. The Q statistic was calculated to estimate the heterogeneity, and *p* ≤ 0.10 was considered statistically significant [66]. We assessed the possibility of publication bias for the studies included in the meta-analyses with Egger’s linear regression test, which was used to quantitatively evaluate the publication asymmetry, and a *p* < 0.05 was set as statistically significant.

If it was infeasible to make a quantitative synthesis and conduct a meta-analysis for a variable, studies that reported the same determinants were grouped, and a narrative approach and descriptive statistics were used to compare their associations with the TR.

## 3. Result

### 3.1. Search Results

**Figure 1:**
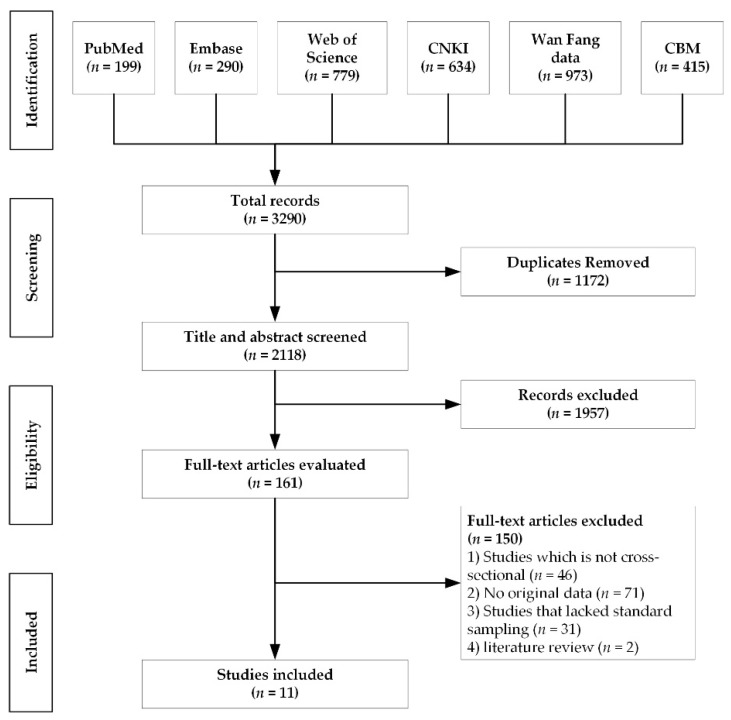
Flow diagram of study selection.

As shown in Figure 1, 3290 articles were initially identified after conducting the search strategy, and 2118 articles remained after directly removing duplicate literature. After reviewing the title or abstract, 1957 articles that did not meet the eligibility criteria were discarded. Among the remaining 161 studies, 150 articles were removed after full-text review for the following reasons: (1) 46 articles were not cross-sectional studies, (2) 71 articles had no original data, (3) 31 articles lacked standard sampling, and (4) 2 articles were literature reviews. Ultimately, 11 studies were included.

### 3.2. Analysis of the Included Articles

The characteristics of the included articles are presented in Table 2. A total of 11 articles contained 65,006 healthcare workers and 31,900 patients distributed throughout China. Of the 11 articles, 4 articles analyzed the determinants only from the healthcare worker perspective (HWP) [67,68,69,70], 2 articles analyzed the determinants from only the patient perspective (PP) [71,72], 1 article analyzed the determinants from both the HWP and the therapeutic interaction perspective (TIP) [73], 2 articles analyzed the determinants from both the PP and the TIP [74,75], 1 article analyzed the determinants from both the HWP and the PP [76], and 1 article analyzed the determinants from the HWP, PP, and TIP [77].

Fifty determinants were extracted from the included studies and were categorized into three groups: (1) 24 healthcare worker-related factors: gender, age, years of experience, education level, professional title, department, hospital type, region, income, income satisfaction, working time per day, marital status, employment form, administrative position, whether disputed with patient, workload, medical ethics, whether medical disputes interfere with work, career satisfaction, ability to handle dispute, whether worry about encountering dispute, daily average rate of outpatient visits, medical liability insurance, and time spent in direct contact with the patient; (2) 18 patient-related factors: gender, age, registered residence, education level, occupation, medical insurance, medical expenses, household income, whether have a familiar doctor, hospital type, region, department, whether first visit, registration, sources of patients, whether have family doctors, referral, and operation; (3) 8 therapeutic interaction-related factors: healthcare worker-patient trust, service attitude, service quality and level, treatment effect, whether healthcare worker receives kickbacks on medications or medical devices, whether adequate medical information is shared, healthcare worker-patient communication, and whether patient bribes or entertains doctors.

### 3.3. Quality of the Included Articles

The quality score of 11 articles ranged from 5 to 7 (shown in Table 3), and the average score was 6 out of 7 according to the modified Newcastle-Ottawa scale. All studies were of good quality. Five articles did not meet the sample representative standard (sample size ≥ 1000). Five articles did not report the non-respondent rate. All studies met other assessment criteria.

### 3.4. Analysis of Healthcare Worker-Related Factors

Of the 11 included articles, a total of 7 analyzed the healthcare worker-related factors. Based on these 7 studies, the incidence of healthcare workers with pessimistic attitudes toward the TR ranged from 8.16% to 82.50%, as shown in Table 2. Overall, the mean proportion of negative attitudes was 55.73% (SE: 10.10%, 95% CI: 31.02–80.43%).

**Figure 2:**
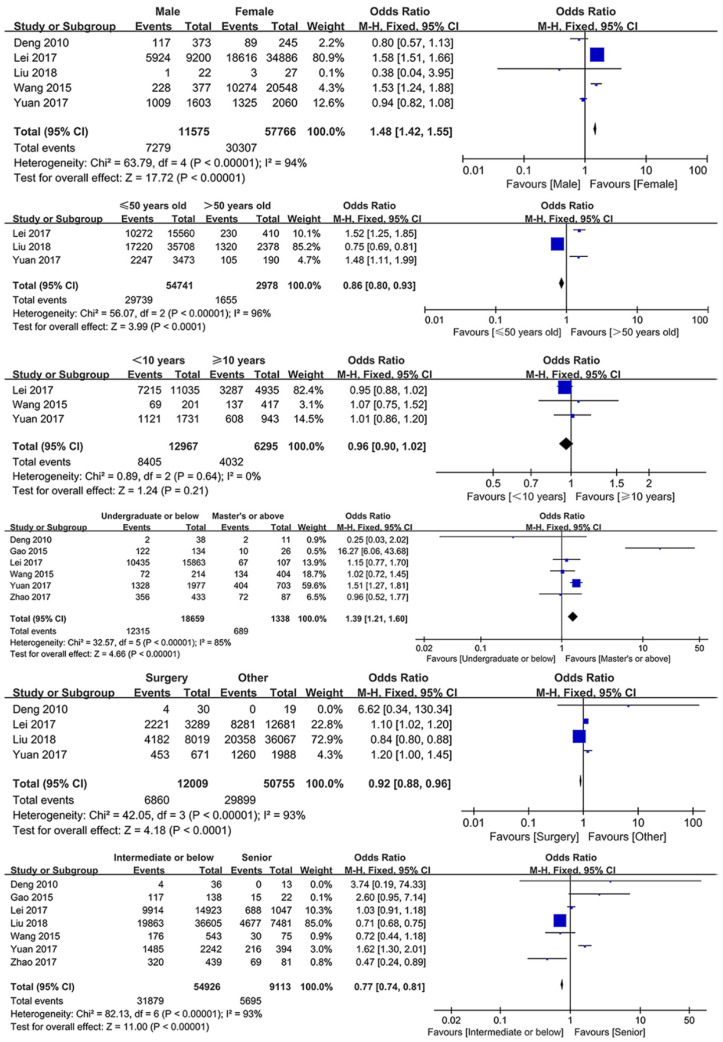
Forest plots of healthcare worker-related determinants.

Among all extracted healthcare worker-related factors, six factors were included in the meta-analysis, and all the extracted determinants were included in the descriptive analysis. Figure 2 shows the meta-analysis results of the healthcare worker-related factors. Gender (male vs. female, OR: 1.48, 95% CI: 1.42–1.55, *p* < 0.00001), age (≤50 years old vs. >50 years old, OR: 0.86, 95% CI: 0.80–0.93, *p* < 0.0001), education level (undergraduate or below vs. master or above, OR: 1.39, 95% CI: 1.21–1.60, *p* < 0.00001), department (surgery vs. other, OR: 0.92, 95% CI: 0.88–0.96, *p* < 0.0001), and professional title (intermediate or below vs. senior, OR: 0.77, 95% CI: 0.74–0.81, *p* < 0.00001) were significantly associated with TR. However, years of experience (*p* = 0.21) was not significantly associated with the TR. The results indicated that male healthcare workers, those over 50 years old, those with a bachelor’s degree or less, those in non-surgical departments and those with senior professional titles were more likely to be pessimistic about the TR.

Among other healthcare worker-related factors reported in these 7 studies, the determinants that were significantly associated with the TR included working time per day, workload, income satisfaction, medical ethics, whether medical disputes interfere with work, whether worry about encountering disputes, daily average rate of outpatient visits, hospital type, administrative position, medical liability insurance, and time spent in direct contact with patients. The occurrence of pessimistic attitudes and medical disputes was more likely for healthcare workers who had longer working times [67,70], had greater working pressure [70,73], were less satisfied with income [73], had worse medical ethics [73], had more daily outpatients [70], worked in comprehensive hospitals rather than specialty hospitals [69], had no administrative position [69], had no medical liability insurance [69], and spent longer time in contact with patients [67]. Meanwhile, the more likely the healthcare workers are to be affected by medical disputes, the more pessimistic their perception of the TR is [73].

### 3.5. Analysis of Patient-Related Factors

Of the 11 included articles, a total of 6 analyzed patient-related factors. Based on the 6 studies, the incidence of patients with pessimistic attitudes toward the TR ranged from 13.19% to 57.10%, as shown in Table 2. Overall, the mean proportion of negative attitudes was 33.45% (SE: 6.70%, 95% CI: 16.24–50.66%).

**Figure 3:**
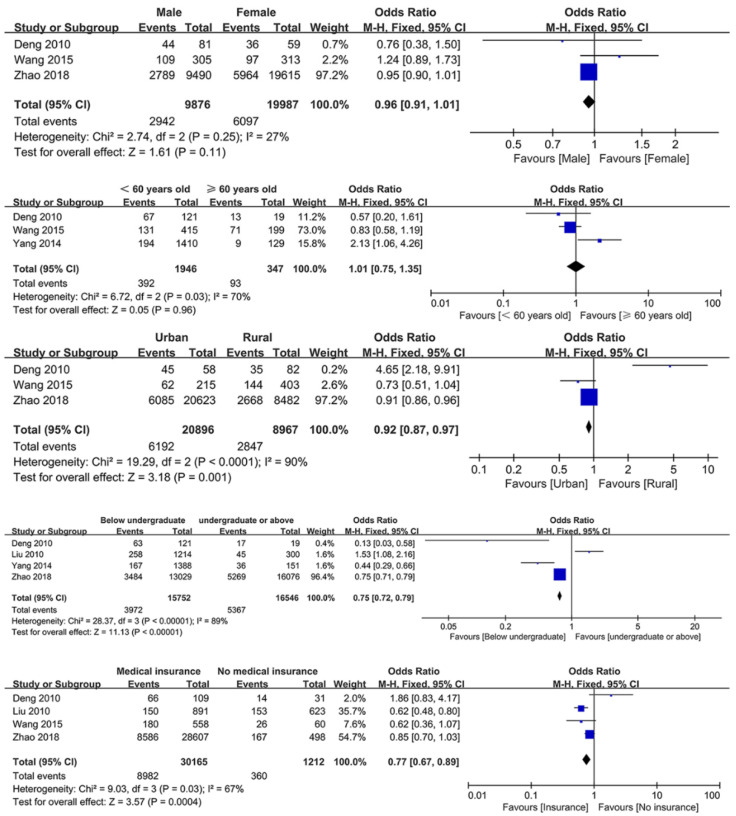
Forest plots of patient-related determinants.

Among all extracted patient-related factors, five determinants were included in the meta-analysis, and all the extracted determinants were included in the descriptive analysis. Figure 3 shows the meta-analysis results of patient-related factors. Registered residence (urban vs. rural, OR: 0.92, 95% CI: 0.87–0.97, *p* < 0.001), education level (below undergraduate vs. undergraduate or above, OR: 0.75, 95% CI: 0.72–079, *p* < 0.00001), and lack of medical insurance (medical insurance vs. no medical insurance, OR: 0.77, 95% CI: 0.67–0.89, *p* = 0.0004) were significantly associated with TR. However, gender (*p* = 0.11) and age (*p* = 0.96) were not significantly associated with the TR. The results indicated that rural patients, patients with a bachelor’s degree or above, and uninsured patients were more likely to be pessimistic about the TR.

Among other patient-related factors reported in these 6 studies, the determinants that were significantly associated with the TR included occupation, medical expenses, household income, whether they had a familiar doctor, type of visiting hospital, region, department, and whether they were at their first visit, and whether they had family doctors. The occurrence of pessimistic attitudes or medical disputes was more likely to occur in patients who had no regular occupation [77], had higher medical expenses [74,77], had no familiar doctors or family doctors [71,74], were not at their first visit [71], visited a specialty hospital [71], visited the department of gynecology or pediatrics [71], and resided in the western region of China [71].

### 3.6. Analysis of Therapeutic Interaction-Related Factors

**Figure 4:**
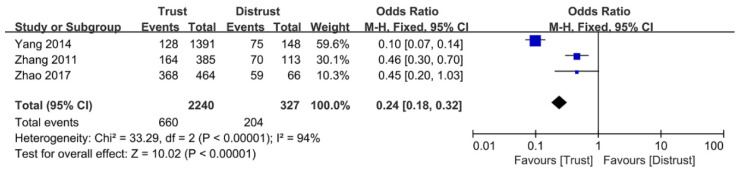
The forest plot of healthcare worker-patient trust.

Of the 11 included articles, a total of 4 analyzed the therapeutic interaction-related factors. Among all extracted therapeutic interaction-related factors, one determinant was included in the meta-analysis, whereas all the extracted determinants were included in the descriptive analysis. Figure 4 shows the meta-analysis results of therapeutic interaction-related factors. Healthcare worker-patient trust (trust vs. distrust, OR: 0.24, 95% CI: 0.18–0.32, *p* < 0.00001) was significantly associated with TR. The results indicated that cultivating trust between healthcare workers and patients can reduce the conflicts between them.

Among other therapeutic interaction-related factors reported in 4 studies, the determinants that were significantly associated with the TR included service attitude, service quality and level, treatment effect, whether healthcare worker receives kickbacks on medications or medical devices, whether adequate medical information is shared, healthcare worker-patient communication, and whether patient bribes or entertains doctors. These 3 of 4 studies reported that if there are good service attitudes [74,77], high service quality and level [74], good treatment effects [74], sufficient medical information sharing [74], and effective communication [75] in the doctor-patient interaction, the TR will tend to be harmonious. However, if the healthcare workers receive kickbacks on medications or medical devices, or the patients bribe or entertain the doctor during the treatment [74], the TR will become tense.

## 4. Discussion

In this study, we conducted a systematic review of the present situation and related determinants of the TR in China. Seven articles reported the present situation of the TR from the perspective of healthcare workers, whereas six articles reported the present situation of the TR from the perspective of patients.

The synthesis of the data of 11 articles confirmed that there were significant differences in the evaluation of the TR situation between healthcare workers and patients [35]. The proportion of healthcare workers with pessimistic attitudes toward the TR ranged from 8.16% to 82.50% (mean: 55.73%, SE: 10.10%, 95% CI: 31.02–80.43%). However, the proportion of patients with pessimistic attitudes toward the TR ranged from 13.19% to 57.10% (mean: 33.45%, SE: 6.70%, 95% CI: 16.24–50.66%). The 95% confidence intervals of two groups are overlapped, which cannot definitively support that the proportion of healthcare workers who held that the TR with a relatively tense situation was higher than that of patients. However, plenty of previous studies testified this finding [35,52,78,79]. This finding was considered to be the result of the poor practice environment and the high sensitivity to disputes of healthcare workers [80,81], which was thought to be related to the fact that most patients are inclined to rely on and trust healthcare workers when seeking medical help [35]. Medical activities account for a large proportion in the life of healthcare workers. For example, the opportunity and time for healthcare workers to participate in healthcare workers-patient interactions are often much higher than that of patients. In addition, healthcare workers in China, especially those in tertiary hospitals, are under a bad practice environment such as heavy workload and working pressure [82], heavy pressure from public opinion [79], and social prejudice [83]. Therefore, healthcare workers have more pressure to maintain the TR than patients, and they are highly sensitive to medical disputes and have a sense of self-protection. In Nigeria, a study showed that the doctor-patient relationship was the most important practice orientation for doctors [39]. Taking defensive medicine for instance [84], in order to avoid lawsuits for misdiagnosis, healthcare workers often perform multiple medical checkups (though some are found unnecessary after a patient’s disease is diagnosed). Obviously, defensive medicine is an important response behavior of healthcare workers with high sensitivity to medical disputes. However, defensive medicine is also one of the reasons for the increase in medical costs in recent years [84], further exacerbating the problem of “expensive to get medical treatment” in China. In the long run, defensive medicine is bound to do more harm than good to the TR. As for the patients, they generally trust and have faith in healthcare workers as a group [85]. Moreover, patients demanding quality care are becoming commonplace [86,87]. The slow pace of the reformation of China’s healthcare system is strong evidence of this phenomenon. In order to alleviate the working pressure of tertiary hospitals, as well as the problem of “difficulty in getting medical treatment,” the Health Ministry of China has been committed to diverting patients’ choice of medical treatment through the implementation of a tiered diagnosis and treatment model since 2015. However, due to the lack of supporting policies and the low quality of primary medical services [88], it is still unable to effectively guide patients with mild illness to seek medical treatment in community hospitals or secondary hospitals. The situation is the opposite of that in the UK in that National Health Service has a strict system of referrals. Except for emergencies, British citizens with general health problems are the first to go to a general practitioner. Most problems can be solved successfully, even if there are a few difficult problems that require a referral to the hospital [89]. Although few studies have been done in other countries on cognitive differences of the TR between healthcare workers and patients, such differences do exist in other countries [90]. For example, a study from Canada reported that due to information asymmetry, differences in the professional division of labor, knowledge background, and differences in rights and interests between healthcare workers and patients, both sides have significantly different cognitions and attitudes toward TR [91]. This study also analyzed the related determinants of the TR. A total of 50 determinants were identified, of which 36 were determined to have significant associations with the TR, including 16 healthcare worker-related factors, 12 patient-related factors, and 8 therapeutic interaction-related factors.

### 4.1. Impact of Healthcare Worker-Related Factors

In terms of healthcare worker-related factors, first, male healthcare workers were more likely to hold a pessimistic attitude toward the TR than female healthcare workers were. However, many previous studies in China reported that there was no gender difference [30,67,68,77]. Conversely, studies in other countries reported the same conclusion as our study [34,36,38,39,40]. Previous studies argued that female doctors had advantages over men in many aspects of medical treatment. For example, female doctors are more patient-oriented [37] and more sensitive to relationship values [92]. Patients generally preferred female doctors because they deemed that the characteristics of female doctors were more in line with the characteristics of “good doctors.” An investigation found that 63% of female doctors felt their relationship with their patients was friendly rather than business-like, compared with only 42% of male doctors [38]. Second, we concluded that healthcare workers over 50 years old were more likely to hold pessimistic attitudes toward TR. In contrast, this finding is inconsistent with the existing evidence from previous studies, which showed that doctors had high risks of holding pessimistic attitudes toward TR were those who were younger (lower age) [28,29,30,31,32,33]. Moreover, Yun X, et al. and Tianjiao M, et al. reported that healthcare workers over 50 years old had a better appraisal of the TR. In Nigeria, doctors aged 30 years and above had significantly higher mean scores on doctor-patient relationships than their colleagues [39]. Third, healthcare workers with a master’s degree or above had a better perception of the TR, partly because healthcare workers with an advanced degree received more empathy and medical humanities education. Although China’s medical humanities education is weak in general [93], previous study has shown that the higher the grade of medical students, the more medical humanities education they receive, and the better their ability of humanistic care [94]. Whereas plenty of studies have proved that empathy and medical humanistic education have a positive effect on TR [95,96,97,98]. A study in the United States also considered that a better education background reflected, to a certain extent, excellent medical skills and flexible communication skills in healthcare workers [43]. Meanwhile, education played a buffering role in avoiding medical disputes as communication skills, doctor-patient relationships and other issues are covered in higher education [43]. Some previous studies reported the same results [68,70]. Fourth, the relationship with patients is more harmonious for surgical healthcare workers than for other departments’ healthcare workers. A previous study found that the proportion of surgeons who believed that the TR was increasingly harmonious was significantly higher than that of practitioners of internal medicine, obstetrics, gynecology, etc., [28]. Meanwhile, some studies concluded that healthcare workers in emergency departments were more likely to experience medical disputes than healthcare workers in other departments [48,49,67,68]. On the one hand, the patients in the emergency department and their family members are more anxious and have higher expectations for the healthcare workers [99]; on the other hand, the healthcare workers in the emergency department have a heavy workload [99]. Thus, it is particularly crucial to cultivate empathy between doctors and patients in the emergency department. Fifth, senior professional titles had a significant association with a high risk of medical disputes. A previous study reported that compared with intermediate and junior doctors, senior doctors had a stronger sense of tension toward the TR [46]. Although these senior doctors had rich experience in communication with patients, their workload and work pressure affected the doctor-patient relationship. In addition to the above factors, substantial previous studies have suggested that doctors with high income-satisfaction could maintain a harmonious relationship with patients [45,52,53,73]. In Germany, America, and Italy, a better healthcare workers-patient relationship was correlated with higher job satisfaction [100,101,102], which was confirmed by many previous studies [29,103,104,105,106,107]. In studies from the United States and Hong Kong, a heavy workload was shown to worsen the quality of communication and medical service, which in turn easily triggered medical disputes [50,108]. In Iran and the United States, a better doctor-patient relationship was inseparable from the enhancement of medical ethics [54,55].

### 4.2. Impact of Patient-Related Factors

In terms of patient-related factors, first, rural patients were more likely to hold a pessimistic attitude toward the TR than urban patients, which was consistent with the conclusions of previous studies in China [41,71,76]. As the majority of urban patients had more income than rural patients, their medical burden was relatively lighter, which partly eased the tensions. Nevertheless, a study from Germany suggested that urban patients showed poorer doctor-patient relationships than rural patients [56]. Second, patients with a better educational background tended to hold a worse perception of TR. This conclusion was similar to that of studies in China [30,41,71] and other countries [57,58]. Patients with low educational levels are highly dependent on and have high trust in doctors [30,57]. In contrast, patients with better educational backgrounds had higher expectations and requirements of doctors in all aspects, which partly raised the bar for patient satisfaction. Finally, medical insurance, medical expense, and household income had significant associations with TR. High medical expenses would place a substantial economic burden on patients with low household incomes, which could increase their dissatisfaction with healthcare workers when defensive medical behavior occurs. Adequate evidence has shown that patients with medical insurance tend to have a better relationship with healthcare workers than do patients who pay out of pocket [41,71,72,76]. Moreover, the reimbursement rate and range of medical insurance coverage may also affect the TR [109]. Therefore, the establishment of a sound medical insurance system, to a certain extent, would ease patient burdens and improve the TR [47,59].

### 4.3. Impact of Therapeutic Interaction-Related Factors

In terms of therapeutic interaction-related factors, first, a higher prevalence of medical disputes was found among the therapeutic relationships with a low degree of trust. Mutual trust between healthcare workers and patients could construct a favorable atmosphere for the TR. Substantial previous studies from Norway [60], Singapore [110], America [61], the Netherlands [111], Australia [112], and China [47,109] argued that trust played a crucial role in fostering a win-win TR. Second, effective communication could promote rapport between healthcare workers and patients. This viewpoint is consistent with abundant previous studies [62,63,113,114,115]. The important components of effective communication should include patient-centered communication [60,116] and adequate medical information sharing [59,117]. Therefore, this study supports the idea that healthcare workers occupy the dominant position in healthcare worker-patient communication, which suggests that training in communication skills for healthcare workers should be strengthened [118,119]. Third, service attitude [39,81] and medical quality [39,120] had significant associations with TR. Evidence from a previous study showed that patients not only paid attention to the quality of their medical results (medical effects) but also took the quality of the service process seriously [121]. Meanwhile, service attitude is also an important factor in the assessment of service process quality. Therefore, more attention to doctors’ behavior and attitudes is needed in the process of standardizing medical services. Finally, the existence of medical rebates and rent-seeking behavior negatively impact TR [46,52,74,122,123,124].

### 4.4. Study Strengths and Limitations

To our knowledge, this is the first systematic review to analyze the determinants related to the TR in China. Our findings present an overview of the current evidence from Mainland China. One strength of this review was that the analysis of the TR in China was based on a large sample size of 11 articles and 96,906 individuals (65,006 healthcare workers and 31,900 patients). Another strength was the comprehensive analyses of the determinants, particularly the meta-analysis and narrative analysis. Fifty determinants were extracted from the 11 included studies. Sixteen healthcare worker-related factors, twelve patient-related factors, and eight therapeutic interaction-related factors were significantly associated with the TR. However, three limitations existed in this systematic review. First, significant heterogeneity among the individual studies was found when performing the meta-analyses, which limited the ability to synthesize evidence clearly. Second, articles presenting results from other health professionals such as pharmacists and technicians have not been obtained. Most empirical studies on TR in China are conducted from the perspectives of doctors and nurses, while few studies on TR among healthcare workers in other professions, among which no relevant studies met the selection criteria. Furthermore, articles using the Patient-Doctor Relationship Questionnaire (PDRQ) and the Difficult Doctor-Patient Relationship Questionnaire (DDPRQ) measurement tools were not included in this study, resulting in that the current situation of the TR in China could not be measured systematically. To address these limitations, more studies should be included by changing the search strategy. Furthermore, additional studies that explore the influences of health policy-related determinants on the TR in the Chinese context are necessary. It would also be meaningful to further review qualitative studies on the TR to add depth and help explain some of the reported findings.

## 5. Conclusions

With a screened sample including 65,006 healthcare workers and 31,900 patients, this systematic review and meta-analysis highlight the attitudes toward TR and the heterogeneity among different groups of people. More than half (55.73%) of healthcare workers and more than one-third of patients (33.70%) believe their relationship is tensed at present. The percentages are significantly higher among those of male, old, less educated, and senior-titled health workers and rural, well-educated, and also those disadvantaged non-insured patients. The findings might deserve consideration in the design of relative policies to ensure harmony between doctors and patients. There is no doubt that strengthening mutual communication and therapeutic relationship strategies based on person-centered care (PCC) is the key to improving the relationship from the perspective of health professionals. Given the current situation of heavy workload in tertiary hospitals in China [125], the government may want to pay more attention to the burnout of health workers in tertiary hospitals.

## Figures and Tables

**Table 1:** Characteristics of selection criteria.

Characteristics	Criteria
Study type	Empirical studies published in a peer-reviewed journal
Study design	Cross-sectional studies
P (Population)	Doctors/Nurses and Patients
I (Intervention)	Population with positive attitudes towards the TR
C (Comparator)	Population with pessimistic attitudes towards the TR
O (Outcomes)	Studies that analysed the current situation and the related determinants, factors or influences of TR
Language	None language restrictions
Publication period	After 1 January 2000 and before 1 January 2020

**Table 2:** Characteristics of 11 included studies.

Authors	Location	Participants	Sample Size (Effective Response Rate %)	Analytical Perspective	Determinants	Negative Cases Rate *, N (%)
Shi, 2017 [67]	16 provinces * (municipalities or autonomous regions)	Nurses	15,970 (74.77)	HWP	Gender, Age, Education level, Marital status, Professional title, Employment form, Income, Department, Years of experience, Working time per day, Time spent in direct contact with patients.	10,502 (65.76)
Zhao, 2017 [73]	Chongqing	Medical personnel	520 (not stated)	HWP & TIP	HWP: Education level, Professional title, Income satisfaction, Medical ethics, Workload, Whether disputed with patient, Whether medical disputes interfere with work. TIP: Healthcare worker-patient trust.	421 (80.96)
Yuan, 2017 [68]	Shanxi	Medical personnel	3663 (81.40)	HWP	Gender, Age, Years of experience, Education level, Professional title, Marital status, Employment form, Department, Income.	2334 (63.72)
Liu, 2018 [69]	Nationwide	Medical personnel	44,086 (not stated)	HWP	Gender, Age, Hospital type, Region, Professional title, Administrative position, Department, Income, Medical liability insurance.	24,540 (55.66)
Gao, 2015 [70]	Chongqing	Doctors	160 (100)	HWP	Education level, Professional title, Workload, Daily average rate of outpatient visits, Working time per day, Whether disputed with patient, Whether worry about encountering dispute.	132 (82.50)
Zhao, 2018 [71]	Nationwide	Outpatients	29,105 (99.82)	PP	Hospital type, Region, Age, Gender, Department, Whether first visit, Education level, Medical insurance, Household income, Registration, Registered residence, Sources of patients, Whether have a familiar doctor, Whether have family doctors, Referral.	8752 (30.07)
Zhang, 2011 [74]	Weifang	Patients	498 (not stated)	PP & TIP	PP: Medical expenses, Whether have family doctors. TIP: Service attitude, Service quality and level, Treatment effect, Whether healthcare worker receives kickbacks on medications or medical devices, Doctor-patient trust, Whether adequate medical information is shared, Whether patient bribes or entertains doctors *.	234 (46.99)
Wang, 2015 [77]	Wuhan	Patients & Medical personnel	Medical personnel: 618 (not stated) Patients: 618 (not stated)	HWP & PP & TIP	HWP: Gender, Age, Years of experience, Education level, Professional title, Career satisfaction, ability to handle dispute. PP: Gender, Registered residence, Age, Education level, Occupation, Medical insurance, operation, Medical expenses. TIP: Service attitude.	Medical personnel: 206 (33.33) Patients: 206 (33.33)
Yang, 2014 [75]	Hubei	Patients	1539 (95.60)	PP & TIP	PP: Age, Education level, Medical insurance. TIP: Healthcare worker-patient communication, Healthcare worker-patient trust.	203 (13.19)
Deng, 2010 [76]	Changsha	Patients & Medical personnel	Medical personnel: 49 Patients: 140 (Total qualified rate: 99.47)	HWP & PP	HWP: Age, Gender, Professional title, Department, Education level. PP: Age, Gender, Education level, Registered residence, Medical insurance, Household income.	Medical personnel: 4 (8.16) Patients: 80 (57.10)
Liu, 2010 [72]	Urumqi	Patients	1514 (not stated)	PP	Medical insurance, Education level	303 (20.01)

* 16 provinces (municipalities or autonomous regions) mean Beijing, Tianjin, Hebei, Shandong, Guangdong, Liaoning, Shanxi, Henan, Anhui, Hunan, Heilongjiang, Ningxia, Shannxi, Gansu, Sichuan, and Chongqing. * ‘negative cases’ means the population with pessimistic attitudes towards the TR. * ‘patient entertains doctors’ means that patients invite the doctors to dinner in order to get an appointment with a better doctor, better care, or some preferential treatments else.

**Table 3:** Assessment of risk of bias.

Authors	Sample Representativeness	Sample Size	Respondent Rate	Ascertainment of the Exposure	Comparability of Subjects in Different Outcome Groups	Outcome Assessment	Appropriate Statistical Test	Total Score
Shi, 2017	1	1	1	1	1	1	1	7
Zhao, 2017	0	1	0	1	1	1	1	5
Yuan, 2017	1	1	1	1	1	1	1	7
Liu, 2018	1	1	0	1	1	1	1	6
Gao, 2015	0	1	1	1	1	1	1	6
Zhao, 2018	1	1	1	1	1	1	1	7
Zhang, 2011	0	1	0	1	1	1	1	5
Wang, 2015	0	1	0	1	1	1	1	5
Yang, 2014	1	1	1	1	1	1	1	7
Deng, 2010	0	1	1	1	1	1	1	6
Liu, 2010	1	1	0	1	1	1	0	5

Notes: Study type: cross-sectional; sore: 1 = achieved, 0 = not achieved.

## Data Availability

No new data were created or analyzed in this study. Data sharing is not applicable to this article.

## Supplementary Materials

The following are available online at https://www.mdpi.com/article/10.3390/ijerph18073460/s1, Supplementary File: The search strategy, extracted factors, the distribution of factors and the extracted data.

## Abbreviations

TR	Therapeutic Relationship
PCC	Person-Centered Care
WB	The World Bank
WHO	The World Health Organization
PCIC	People-Centered and Integrated Health Care
PRISMA	Preferred Reporting Items for Systematic Reviews and Meta-Analyses
CNKI	China National Knowledge Internet Database
CBM	China Biology Medicine disc
GRADE	The Grading of Recommendations: Assessment, Development, and Evaluation
HWP	Healthcare worker Perspective
PP	Patient Perspective
TIP	Therapeutic Interaction Perspective
OR	Odds Ratio
PDRQ	Patient-Doctor Relationship Questionnaire
DDPRQ	Difficult Doctor-Patient Relationship Questionnaire
