# Supplementary material for: The Therapeutic Relationship in China: A Systematic Review and Meta-Analysis
Source: Int J Environ Res Public Health. 2021 Mar 26;18(7):3460. doi: 10.3390/ijerph18073460 (PMC8037362; doi:10.3390/ijerph18073460)
Supplement: Supplementary file 1 [file ijerph-18-03460-s001.pdf]

International Journal of
Environmental Research and Public Health

MDPI

Supplementary Materials
1. The Search Strategy

Key word

China/Chinese

Doctor-patient relationship/ Patient-doctor relationship/ Clinician-patient relationship/ Patient-clinician relationship/ Therapist-patient relationship/ Patient-therapist relationship/ Physician-patient relationship/ Patient-Physician relationship/ Doctor-patient relation/ Patient-doctor relation/ Clinician-patient relation/ Patient-clinician relation/ Therapist-patient relation/ Patient-therapist relation/ Physician-patient relation/ Patient-physician relation/ The relationship between doctor and patient/ The relationship between clinician and patient/ The relationship between physician and patient/The relation between doctor and patient/ The relation between clinician and patient/ The relation between physician and patient/ The interactions between doctor and patient/ The interactions between clinician and patient/ The interactions between physician and patient/ Medical dispute/ Conflict between doctor and patient/ Conflict between physician and patient/ Conflict between clinician and patient/ Nurse-patient relationship/ Medical trouble

Determinants/ Factors/ Influences

中国、中国的

医患矛盾、医患关系、医患纠纷、医疗纠纷、医闹

影响因素、成因

Search strategy（Publication date: Jan. 2000 to Jan. 2020）

Database 1: CAJD (CNKI)

SU=（‘医患矛盾’+‘医患关系’+‘医患纠纷’+‘医疗纠纷’+‘医闹’） AND SU=（‘影响因素’+‘成因’）

Search Results：634

Database 2: CSPD (WANFANG Data)

(题名或关键词:(医患矛盾)+题名或关键词:(医患关系)+题名或关键词:(医患纠纷)+题名或关键词:(医疗纠纷)+题名或关键词:(医闹)) and (题名或关键词:(影响因素)+题名或关键词:(成因))

Search Results：973

Database 3: CBM（SinoMed）

((((("医患矛盾"[标题:智能]) OR "医患关系"[标题:智能]) OR "医患纠纷"[标题:智能]) OR "医疗纠纷"[标题:智能]) OR "医闹"[标题:智能]) AND ("影响因素"[标题:智能] OR ("成因"[标题:智能]))

Search Results：415

Table S1. Database 4: PubMed (NCBI)

Search	Query	Items Found
#1	Search (China[MeSH Terms]) OR China[Title/Abstract]	251,210
#2	Search (Chinese[MeSH Terms]) OR Chinese[Title/Abstract]	242,638
#3	Search (((China[MeSH Terms]) OR China[Title/Abstract])) OR ((Chinese[MeSH Terms]) OR Chinese[Title/Abstract])	416,173
#4	Search (Doctor-patient relationship[MeSH Terms]) OR Doctor-patient relationship[Title/Abstract]	3359
#5	Search (Patient-doctor relationship[MeSH Terms]) OR Patient-doctor relationship[Title/Abstract]	505
#6	Search (Clinician-patient relationship[MeSH Terms]) OR Clinician-patient relationship[Title/Abstract]	152
#7	Search (Patient-clinician relationship[MeSH Terms]) OR Patient-clinician relationship[Title/Abstract]	115
#8	Search (Therapist-patient relationship[MeSH Terms]) OR Therapist-patient relationship[Title/Abstract]	87
#9	Search (Patient-therapist relationship[MeSH Terms]) OR Patient-therapist relationship[Title/Abstract]	104
#10	Search (Physician-patient relationship[MeSH Terms]) OR Physician-patient relationship[Title/Abstract]	72,201
#11	Search (Patient-physician relationship[MeSH Terms]) OR Patient-physician relationship[Title/Abstract]	71,999
#12	Search (Doctor-patient relation[MeSH Terms]) OR Doctor-patient relation[Title/Abstract]	71,557
#13	Search (Patient-doctor relation[MeSH Terms]) OR Patient-doctor relation[Title/Abstract]	41
#14	Search (Clinician-patient relation[MeSH Terms]) OR Clinician-patient relation[Title/Abstract]	41

#15	Search (Patient-clinician relation[MeSH Terms]) OR Patient-clinician relation[Title/Abstract]	35
#16	Search (Therapist-patient relation[MeSH Terms]) OR Therapist-patient relation[Title/Abstract]	18
#17	Search (Patient-therapist relation[MeSH Terms]) OR Patient-therapist relation[Title/Abstract]	19
#18	Search (Physician-patient relation[MeSH Terms]) OR Physician-patient relation[Title/Abstract]	71,547
#19	Search (Patient-physician relation[MeSH Terms]) OR Patient-physician relation[Title/Abstract]	6087
#20	Search (The relationship between doctor and patient[MeSH Terms]) OR The relationship between doctor and patient[Title/Abstract]	876
#21	Search (The relationship between clinician and patient[MeSH Terms]) OR The relationship between clinician and patient[Title/Abstract]	55
#22	Search (The relationship between physician and patient[MeSH Terms]) OR The relationship between physician and patient[Title/Abstract]	10,895
#23	Search (The relation between doctor and patient[MeSH Terms]) OR The relation between doctor and patient[Title/Abstract]	1423
#24	Search (The relation between clinician and patient[MeSH Terms]) OR The relation between clinician and patient[Title/Abstract]	106
#25	Search (The relation between physician and patient[MeSH Terms]) OR The relation between physician and patient[Title/Abstract]	16,531
#26	Search (The interactions between doctor and patient[MeSH Terms]) OR The interactions between doctor and patient[Title/Abstract]	153
#27	Search (The interactions between clinician and patient[MeSH Terms]) OR The interactions between clinician and patient[Title/Abstract]	20
#28	Search (The interactions between physician and patient[MeSH Terms]) OR The interactions between physician and patient[Title/Abstract]	1899

#29	Search (Medical dispute[MeSH Terms]) OR Medical dispute[Title/Abstract]	2004
#30	Search (Conflict between doctor and patient[MeSH Terms]) OR Conflict between doctor and patient[Title/Abstract]	127
#31	Search (Conflict between physician and patient[MeSH Terms]) OR Conflict between physician and patient[Title/Abstract]	1766
#32	Search (Conflict between clinician and patient[MeSH Terms]) OR Conflict between clinician and patient[Title/Abstract]	13
#33	Search (Medical trouble[MeSH Terms]) OR Medical trouble[Title/Abstract]	1882
#34	Search (Nurse-patient relationship[MeSH Terms]) OR Nurse-patient relationship[Title/Abstract]	19,898
#35	Search (((((((((((((((((((((((((((((((Doctor-patient relationship[MeSH Terms]) OR Doctor-patient relationship[Title/Abstract])) OR ((Patient-doctor relationship[MeSH Terms]) OR Patient-doctor relationship[Title/Abstract])) OR ((Clinician-patient relationship[MeSH Terms]) OR Clinician-patient relationship[Title/Abstract])) OR ((Patient-clinician relationship[MeSH Terms]) OR Patient-clinician relationship[Title/Abstract])) OR ((Therapist-patient relationship[MeSH Terms]) OR Therapist-patient relationship[Title/Abstract])) OR ((Patient-therapist relationship[MeSH Terms]) OR Patient-therapist relationship[Title/Abstract])) OR ((Physician-patient relationship[MeSH Terms]) OR Physician-patient relationship[Title/Abstract])) OR ((Patient-physician relationship[MeSH Terms]) OR Patient-physician relationship[Title/Abstract])) OR ((Doctor-patient relation[MeSH Terms]) OR Doctor-patient relation[Title/Abstract])) OR ((Patient-doctor relation[MeSH Terms]) OR Patient-doctor relation[Title/Abstract])) OR ((Clinician-patient relation[MeSH Terms]) OR Clinician-patient	121,481

	relation[Title/Abstract])) OR ((Patient-clinician relation[MeSH Terms]) OR Patient-clinician relation[Title/Abstract])) OR ((Therapist-patient relation[MeSH Terms]) OR Therapist-patient relation[Title/Abstract])) OR ((Patient-therapist relation[MeSH Terms]) OR Patient-therapist relation[Title/Abstract])) OR ((Physician-patient relation[MeSH Terms]) OR Physician-patient relation[Title/Abstract])) OR ((Patient-physician relation[MeSH Terms]) OR Patient-physician relation[Title/Abstract])) OR ((The relationship between doctor and patient[MeSH Terms]) OR The relationship between doctor and patient[Title/Abstract])) OR ((The relationship between clinician and patient[MeSH Terms]) OR The relationship between clinician and patient[Title/Abstract])) OR ((The relationship between physician and patient[MeSH Terms]) OR The relationship between physician and patient[Title/Abstract])) OR ((The relation between doctor and patient[MeSH Terms]) OR The relation between doctor and patient[Title/Abstract])) OR ((The relation between clinician and patient[MeSH Terms]) OR The relation between clinician and patient[Title/Abstract])) OR ((The relation between physician and patient[MeSH Terms]) OR The relation between physician and patient[Title/Abstract])) OR ((The interactions between doctor and patient[MeSH Terms]) OR The interactions between doctor and patient[Title/Abstract])) OR ((The interactions between clinician and patient[MeSH Terms]) OR The interactions between clinician and patient[Title/Abstract])) OR ((The interactions between physician and patient[MeSH Terms]) OR The interactions between physician and patient[Title/Abstract])) OR ((Medical	

	dispute[MeSH Terms]) OR Medical dispute[Title/Abstract])) OR ((conflict between doctor and patient[MeSH Terms]) OR conflict between doctor and patient[Title/Abstract])) OR ((conflict between physician and patient[MeSH Terms]) OR conflict between physician and patient[Title/Abstract])) OR ((conflict between clinician and patient[MeSH Terms]) OR conflict between clinician and patient[Title/Abstract])) OR ((Medical trouble[MeSH Terms]) OR Medical trouble[Title/Abstract])) OR ((Nurse-patient relationship[MeSH Terms]) OR Nurse-patient relationship[Title/Abstract])	
#36	Search (Determinants[MeSH Terms]) OR Determinants[Title/Abstract]	153,077
#37	Search (factors[MeSH Terms]) OR factors[Title/Abstract]	1,998,025
#38	Search (influences[MeSH Terms]) OR influences[Title/Abstract]	197,020
#39	Search ((((Determinants[MeSH Terms]) OR Determinants[Title/Abstract])) OR ((factors[MeSH Terms]) OR factors[Title/Abstract])) OR ((influences[MeSH Terms]) OR influences[Title/Abstract])	2,264,083
#40	Search ((((((China[MeSH Terms]) OR China[Title/Abstract])) OR ((Chinese[MeSH Terms]) OR Chinese[Title/Abstract]))) AND (((((((((((((((((((((((((((((((((Doctor-patient relationship[MeSH Terms]) OR Doctor-patient relationship[Title/Abstract])) OR ((Patient-doctor relationship[MeSH Terms]) OR Patient-doctor relationship[Title/Abstract])) OR ((Clinician-patient relationship[MeSH Terms]) OR Clinician-patient relationship[Title/Abstract])) OR ((Patient-clinician relationship[MeSH Terms]) OR Patient-clinician relationship[Title/Abstract])) OR ((Therapist-patient relationship[MeSH Terms]) OR Therapist-patient relationship[Title/Abstract])) OR ((Patient-therapist relationship[MeSH Terms]) OR Patient-therapist relationship[Title/Abstract])) OR	213

	((Physician-patient relationship[MeSH Terms]) OR Physician-patient relationship[Title/Abstract])) OR ((Patient-physician relationship[MeSH Terms]) OR Patient-physician relationship[Title/Abstract])) OR ((Doctor-patient relation[MeSH Terms]) OR Doctor-patient relation[Title/Abstract])) OR ((Patient-doctor relation[MeSH Terms]) OR Patient-doctor relation[Title/Abstract])) OR ((Clinician-patient relation[MeSH Terms]) OR Clinician-patient relation[Title/Abstract])) OR ((Patient-clinician relation[MeSH Terms]) OR Patient-clinician relation[Title/Abstract])) OR ((Therapist-patient relation[MeSH Terms]) OR Therapist-patient relation[Title/Abstract])) OR ((Patient-therapist relation[MeSH Terms]) OR Patient-therapist relation[Title/Abstract])) OR ((Physician-patient relation[MeSH Terms]) OR Physician-patient relation[Title/Abstract])) OR ((Patient-physician relation[MeSH Terms]) OR Patient-physician relation[Title/Abstract])) OR ((The relationship between doctor and patient[MeSH Terms]) OR The relationship between doctor and patient[Title/Abstract])) OR ((The relationship between clinician and patient[MeSH Terms]) OR The relationship between clinician and patient[Title/Abstract])) OR ((The relationship between physician and patient[MeSH Terms]) OR The relationship between physician and patient[Title/Abstract])) OR ((The relation between doctor and patient[MeSH Terms]) OR The relation between doctor and patient[Title/Abstract])) OR ((The relation between clinician and patient[MeSH Terms]) OR The relation between clinician and patient[Title/Abstract])) OR ((The relation between physician and patient[MeSH Terms]) OR The relation between physician and	

	patient[Title/Abstract])) OR ((The interactions between doctor and patient[MeSH Terms]) OR The interactions between doctor and patient[Title/Abstract])) OR ((The interactions between clinician and patient[MeSH Terms]) OR The interactions between clinician and patient[Title/Abstract])) OR ((The interactions between physician and patient[MeSH Terms]) OR The interactions between physician and patient[Title/Abstract])) OR ((Medical dispute[MeSH Terms]) OR Medical dispute[Title/Abstract])) OR ((conflict between doctor and patient[MeSH Terms]) OR conflict between doctor and patient[Title/Abstract])) OR ((conflict between physician and patient[MeSH Terms]) OR conflict between physician and patient[Title/Abstract])) OR ((conflict between clinician and patient[MeSH Terms]) OR conflict between clinician and patient[Title/Abstract])) OR ((Medical trouble[MeSH Terms]) OR Medical trouble[Title/Abstract])) OR ((Nurse-patient relationship[MeSH Terms]) OR Nurse-patient relationship[Title/Abstract]))) AND (((((Determinants[MeSH Terms]) OR Determinants[Title/Abstract])) OR ((factors[MeSH Terms]) OR factors[Title/Abstract])) OR ((influences[MeSH Terms]) OR influences[Title/Abstract]))	
Studies with publication date from Jan.1.2000 to Jan.1.2020 are included.		199

MeSH (Medical Subject Headings) is the NLM (National Library Of Medicine) controlled vocabulary thesaurus used for indexing articles for PubMed.

Table S2. Database 5: EMBASE.

No.	Query	Results
#1	('china':ti,ab,kw OR 'china'/exp) AND [2000–2020]/py	256,538
#2	('chinese':ti,ab,kw OR 'chinese'/exp) AND [2000–2020]/py	234,849
#3	#1 OR #2	423,407
#4	('Doctor-patient relationship':ti,ab,kw OR 'Doctor-patient relationship'/exp) AND [2000–2020]/py	84,896
#5	('Patient-doctor relationship':ti,ab,kw OR 'Patient-doctor relationship'/exp) AND [2000–2020]/py	84,245
#6	'clinician-patient relationship':ti,ab,kw AND [2000–2020]/py	163
#7	'patient-clinician relationship':ti,ab,kw AND [2000–2020]/py	139
#8	('Therapist-patient relationship':ti,ab,kw OR 'Therapist-patient relationship'/exp) AND [2000–2020]/py	84,132
#9	('Patient-therapist relationship':ti,ab,kw OR 'Patient-therapist relationship'/exp) AND [2000–2020]/py	84,139
#10	('Physician-patient relationship':ti,ab,kw OR 'Physician-patient relationship'/exp) AND [2000–2020]/py	84,546
#11	('Patient-Physician relationship':ti,ab,kw OR 'Patient-Physician relationship'/exp) AND [2000–2020]/py	84,409
#12	('Doctor-patient relation':ti,ab,kw OR 'Doctor-patient relation'/exp) AND [2000–2020]/py	84,111
#13	('Patient-doctor relation':ti,ab,kw OR 'Patient-doctor relation'/exp) AND [2000–2020]/py	84,102
#14	'clinician-patient relation':ti,ab,kw AND [2000–2020]/py	2
#15	'patient-clinician relation':ti,ab,kw AND [2000–2020]/py	2
#16	('Therapist-patient relation':ti,ab,kw OR 'Therapist-patient relation'/exp) AND [2000–2020]/py	84,098
#17	('Patient-therapist relation':ti,ab,kw OR 'Patient-therapist relation'/exp) AND [2000–2020]/py	84,101

#18	('Physician-patient relation':ti,ab,kw OR 'Physician-patient relation'/exp) AND [2000–2020]/py	84,128
#19	('Patient-physician relation':ti,ab,kw OR 'Patient-physician relation'/exp) AND [2000–2020]/py	84,105
#20	'the relationship between doctor and patient':ti,ab,kw AND [2000–2020]/py	43
#21	'the relationship between clinician and patient':ti,ab,kw AND [2000–2020]/py	5
#22	'the relationship between physician and patient':ti,ab,kw AND [2000–2020]/py	31
#23	'the relation between doctor and patient':ti,ab,kw AND [2000–2020]/py	9
#24	'the relation between clinician and patient':ti,ab,kw AND [2000–2020]/py	0
#25	'the relation between physician and patient':ti,ab,kw AND [2000–2020]/py	7
#26	'the interactions between doctor and patient':ti,ab,kw AND [2000–2020]/py	0
#27	'the interactions between clinician and patient':ti,ab,kw AND [2000–2020]/py	0
#28	'the interactions between physician and patient':ti,ab,kw AND [2000–2020]/py	0
#29	'medical dispute':ti,ab,kw AND [2000–2020]/py	88
#30	'conflict between doctor and patient':ti,ab,kw AND [2000–2020]/py	2
#31	'conflict between physician and patient':ti,ab,kw AND [2000–2020]/py	2
#32	'conflict between clinician and patient':ti,ab,kw AND [2000–2020]/py	0
#33	'medical trouble':ti,ab,kw AND [2000–2020]/py	4
#34	'nurse-patient relationship':ti,ab,kw AND [2000–2020]/py	662
#35	#4 OR #5 OR #6 OR #7 OR #8 OR #9 OR #10 OR #11 OR #12 OR #13 OR #14 OR #15 OR #16 OR #17 OR #18 OR #19 OR #20 OR #21 OR #22 OR #23 OR #24 OR #25 OR #26 OR #27 OR #28 OR #29 OR #30 OR #31 OR #32 OR #33 OR #34	86,177
#36	'determinants':ti,ab,kw AND [2000–2020]/py	139,854
#37	'factors':ti,ab,kw AND [2000–2020]/py	2,162,156
#38	'influences':ti,ab,kw AND [2000–2020]/py	193,256
#39	#36 OR #37 OR #38	2,407,086
#40	#3 AND #35 AND #39	290

Table S3. Database 6: Web of Science（Publication Year: 2000–2020)

No.	Query	Results
#1	TS = China	1,069,687
#2	TS = Chinese	885,620
#3	#1 OR #2	1,747,316
#4	TS = Doctor-patient relationship	4795
#5	TS = Patient-doctor relationship	875
#6	TS = Clinician-patient relationship	371
#7	TS = Patient-clinician relationship	419
#8	TS = Therapist-patient relationship	163
#9	TS = Patient-therapist relationship	229
#10	TS = Physician-patient relationship	9794
#11	TS = Patient-Physician relationship	2208
#12	TS = Doctor-patient relation	3976
#13	TS = Patient-doctor relation	746
#14	TS = Clinician-patient relation	380
#15	TS = Patient-clinician relation	464
#16	TS = Therapist-patient relation	120
#17	TS = Patient-therapist relation	202
#18	TS = Physician-patient relation	42,301
#19	TS = Patient-physician relation	2369
#20	TS = (The relationship between doctor and patient)	6012
#21	TS = (The relationship between clinician and patient)	7211
#22	TS = (The relationship between physician and patient)	15,004
#23	TS = (The relation between doctor and patient)	5318
#24	TS = (The relation between clinician and patient)	3572
#25	TS = (The relation between physician and patient)	14,193
#26	TS = (The interactions between doctor and patient)	2345
#27	TS = (The interactions between clinician and patient)	2774
#28	TS = (The interactions between physician and patient)	5615
#29	TS=Medical dispute	5030
#30	TS = (Conflict between doctor and patient)	893
#31	TS = (Conflict between physician and patient)	2208
#32	TS = (Conflict between clinician and patient)	893
#33	TS = medical trouble	6903
#34	TS = Nurse-patient relationship	3751
#35	#4 OR #5 OR #6 OR #7 OR #8 OR #9 OR #10 OR #11 OR #12 OR #13 OR #14 OR #15 OR #16 OR #17 OR #18 OR #19 OR	91,769

	#20 OR #21 OR #22 OR #23 OR #24 OR #25 OR #26 OR #27 OR #28 OR #29 OR #30 OR #31 OR #32 OR #33 OR #34	
#36	TS = Determinants	370,301
#37	TS = Factors	7,528,409
#38	TS = Influences	3,876,183
#39	#36 OR #37 OR #38	10,555,251
#40	#3 AND #35 AND #39	779

TS means ‘theme’ or ‘subject’ and used for indexing articles for Web of Science.

2. The Extracted Determinants

Table S4. 50 determinants

Group	Category	Determinant
A	Healthcare worker-related factors	A1-gender, A2-age, A3-years of experience, A4-education level, A5-professional title, A6-department, A7-hospital type, A8-region, A9-income, A10-income satisfaction, A11-working time per day, A12-marital status, A13-employment form, A14-administrative position, A15-whether disputed with patient, A16-workload, A17-medical ethics, A18-whether medical disputes interfere with work, A19-career satisfaction, A20-ability to handle dispute, A21-whether worry about encountering dispute, A22-daily average rate of outpatient visits, A23-medical liability insurance, A24-time spent in direct contact with the patient
B	Patient-related factors	B1-gender, B2-age, A3-registered residence, B4-education level, B5-occupation, B6-medical insurance, B7-medical expenses, B8-household income, B9-whether have a familiar doctor, B10-hospital type, B11-region, B12-department, B13-whether first visit, B14-registration, B15-sources of patients, B16-whether have family doctors, B17-referral, B18-operation
C	Therapeutic interaction-related factors	C1-health worker-patient trust, C2-service attitude, C3-service quality and level, C4-treatment effect, C5-whether health worker receives kickbacks on medications or medical devices, C6-whether adequate medical information is shared, C7-Health worker-patient communication, C8-whether patient bribes or entertains doctors

3. The Distribution of Determinants

Table S5. Distribution of Determinants in 11 Included Studies (1 = Yes, space = No)

Studies / Exposure	1	2	3	4	5	6	7	8	9	10	11	Total
	Shi, 2017	Zhao, 2017	Yuan, 2017	Liu, 2018	Gao, 2015	Zhao, 2018	Zhang, 2011	Wang, 2015	Yang, 2014	Deng, 2010	Liu, 2010	
A1	1		1	1				1		1		5
A2	1		1	1				1		1		5
A3	1		1					1				3
A4	1	1	1		1			1		1		6
A5	1	1	1	1	1			1		1		7
A6	1		1	1						1		4
A7				1								1
A8				1								1
A9	1		1	1								3
A10		1										1
A11	1				1							2
A12	1		1									2
A13	1		1									2
A14				1								1
A15		1			1							2
A16		1			1							2
A17		1										1
A18		1										1
A19								1				1
A20								1				1
A21					1							1
A22					1							1
A23				1								1
A24	1											1

B1						1		1		1		3
B2						1		1	1	1		4
B3						1		1		1		3
B4						1		1	1	1	1	5
B5								1				1
B6						1		1	1	1	1	5
B7							1	1				2
B8						1				1		2
B9						1	1					2
B10						1						1
B11						1						1
B12						1						1
B13						1						1
B14						1						1
B15						1						1
B16						1						1
B17						1						1
B18								1				1
C1		1					1		1			3
C2							1	1				2
C3							1					1
C4							1					1
C5							1					1
C6							1					1
C7									1			1
C8							1					1
Total	11	8	9	9	7	15	9	16	5	11	2	102

4. The Extracted Data

Data Extraction According to Each Determinant

PERSPECTIVE OF HEALTH WORKER

Table S6. A1-Gender

Author	Group	No Dispute/ Harmony	Dispute/ Tension	Results
Wang, 2015	Male<br>Female	256<br>156	117<br>89	No significant difference (χ^2 test = 1.637, p = 0.201)
Liu, 2018	Male<br>Female	6552<br>32,540	11,848<br>37,232	Significant difference (χ^2 test-p<0.05)
Deng, 2010	Male<br>Female	21<br>24	1<br>3	No significant difference (χ^2 test-p>0.05)
Shi, 2017	Male<br>Female	298<br>10,638	456<br>20,548	No significant difference (χ^2 test-p>0.05)
Yuan, 2017	Male<br>Female	594<br>735	1009<br>1325	No significant difference (χ^2 test = 0.1041, p = 0.7469)

Table S7. A2-Age

Author	Group	No Dispute/ Harmony	Dispute/ Tension	Results
Wang, 2015	<35<br>35–45<br>>45	115<br>232<br>65	63<br>101<br>42	No significant difference (χ^2 test = 3.378, p = 0.185)
Liu, 2018	≤35<br>36–50<br>≥51<br>missing	13,363<br>5125<br>1058<br>6,000	9343<br>7877<br>1320	Significant difference (χ^2 test-p<0.05)
Deng, 2010	<40<br>>40	43<br>2	4<br>0	No significant difference (χ^2 test-p >0.05)
Shi, 2017	≤30<br>31–50<br>≥51	3202<br>2086<br>180	5962<br>4310<br>230	Significant difference (χ^2 test-p<0.001)
Yuan, 2017	≤20<br>20–30<br>30–40<br>40–50<br>>50	37<br>533<br>391<br>265<br>85	92<br>956<br>830<br>369<br>105	No significant difference (χ^2 test = 8.936, p = 0.063)

Table S8. A3-Years of Experience

Author	Group	No Dispute/ Harmony	Dispute/ Tension	Results
Wang, 2015	<10<br>10–20<br>>20	132<br>227<br>53	69<br>102<br>35	No significant difference (χ^2 test = 8.936, p = 0.063)
Shi, 2017	1–4<br>5–10<br>11–20<br>≥21	2488<br>1332<br>814<br>834	4147<br>3068<br>1819<br>1468	Significant difference (χ^2 test-p<0.001)
Yuan, 2017	<5<br>5–9<br>10–15<br>>15	405<br>205<br>102<br>233	659<br>462<br>194<br>414	Significant difference (χ^2 test = 9.8704, p = 0.0197)

Table S9. A4-Education Level

Author	Group	No Dispute/ Harmony	Dispute/ Tension	Results
Zhao, 2017	Master or above<br>Undergraduate<br>Junior college<br>Senior high school or below	15<br>51<br>22<br>4	72<br>307<br>47<br>2	Significant difference (χ^2 test=15.270, p<0.05)
Wang, 2015	Undergraduate<br>Master<br>Doctor	142<br>222<br>48	72<br>113<br>21	No significant difference (χ^2 test = 0.294, p = 0.863)
Gao, 2015	Junior college or below<br>Undergraduate<br>Master or above	2<br>10<br>16	8<br>114<br>10	Significant difference (χ^2 test = 42.615, p = 0.000)
Deng, 2010	Undergraduate<br>Master<br>Doctor	36<br>4<br>5	2<br>2<br>0	Significant difference (χ^2 test-p <0.05)
Shi, 2017	Below undergraduate<br>Undergraduate<br>Master's or above	2625<br>2803<br>40	4665<br>5770<br>67	Significant difference (χ^2 test-p<0.05)
Yuan, 2017	Junior college or below<br>Undergraduate<br>Master or above	122<br>527<br>299	237<br>1091<br>404	Significant difference (χ^2 test = 15.751, p = 0.001)

Table S10. A5-Professional Title

Author	Group	No Dispute/ Harmony	Dispute/ Tension	Results
Zhao, 2017	Senior	12	69	Significant deference

	Intermediate<br>Junior<br>None professional title	45<br>55<br>19	133<br>164<br>23	(χ^2 test = 13.107, p<0.05)
Wang, 2015	Junior<br>Intermediate<br>Senior	136<br>231<br>45	68<br>108<br>30	No significant difference (χ^2 test = 1.832, p = 0.400)
Gao, 2015	Junior<br>Intermediate<br>Senior	12<br>9<br>7	68<br>49<br>15	No significant difference (χ^2 test = 3.628, p = 0.163)
Liu, 2018	Junior<br>Intermediate<br>Senior	11,350<br>5392<br>2804	11,862<br>8001<br>4677	Significant difference (χ^2 test = 13.107, p<0.05) Intermediate vs. Junior (OR = 0.886, 95%CI: 0.814–0.965)
Deng, 2010	Junior<br>Intermediate<br>Senior	17<br>15<br>13	4<br>0<br>0	Significant difference (χ^2 test = 8.376, p = 0.016)
Shi, 2017	Junior<br>Intermediate<br>Senior	3855<br>1154<br>359	7239<br>2675<br>688	Significant difference in the tertiary hospitals (χ^2 test = 6.6, p<0.05); No significant difference in county–level hospitals (χ^2 test = 1.5, p>0.05)
Yuan, 2017	Junior<br>Intermediate<br>Senior	560<br>197<br>178	1063<br>422<br>216	Significant difference (χ^2 test = 20.468, p = 0.000)

Table S11. A6-Department

Author	Group	No Dispute/ Harmony	Dispute/ Tension	Results
Liu, 2018	Internal medicine<br>Surgery<br>Gynaecology and obstetrics<br>Paediatrics<br>Emergency<br>Traditional Chinese Medicine<br>Other	4641<br>3837<br>2990<br>1521<br>699<br>321<br>5537	5859<br>4182<br>3510<br>2335<br>1038<br>445<br>7171	Significant difference (χ^2 test-p<0.05) Internal medicine vs. Other (OR = 0.871, 95%CI:0.798–0.952)
Deng, 2010	Surgery<br>non-surgical department	26<br>19	4<br>0	Significant difference (χ^2 test-p<0.05)
Shi, 2017	Emergency<br>Internal medicine<br>Surgery<br>Gynaecology and obstetrics<br>Paediatrics<br>Other	189<br>1629<br>1068<br>361<br>309<br>1912	847<br>3183<br>2221<br>675<br>834<br>2742	Significant difference (χ^2 test-p<0.001) Surgery in tertiary hospitals vs. Other department in tertiary

				hospitals (OR = 1.341, 95%CI: 1.187–1.514)
Yuan, 2017	Outpatient Emergency<br>Internal medicine<br>Surgery<br>Gynaecology and obstetrics<br>Paediatrics<br>Ophthalmology&<br>Otorhinolaryngology<br>ICU<br>Other	19<br>19<br>376<br>218<br>69<br>27<br>22<br>21<br>175	60<br>168<br>626<br>453<br>94<br>48<br>56<br>32<br>176	Significant difference (χ^2 test = 98.409, p = 0.0000) Internal medicine vs. Outpatient (OR = 0.540, 95%CI: 0.30–0.98)

Table S12. A7-Hospital Type

Author	Group	No Dispute/ Harmony	Dispute/ Tension	Results
Liu, 2018	The general hospital<br>The traditional Chinese medicine hospital<br>The women and children's hospital<br>Other specialized hospitals	8858<br>4302<br>4253<br>2133	10,781<br>5349<br>5417<br>2993	Significant difference (χ^2 test-p<0.05) The traditional Chinese medicine hospital vs. Other specialized hospitals (OR = 1.257, 95%CI:1.096–1.441)

Table S13. A8-Region

Author	Group	No Dispute/ Harmony	Dispute/ Tension	Results
Liu, 2018	The eastern region<br>The central region<br>The western region	8394<br>4565<br>6587	10,371<br>5362<br>8807	Significant difference (χ^2 test-p<0.05) The western region vs. The western region (OR = 1.126, 95%CI: 1.035–1.224)

Table S14. A9-Income

Author	Group	No Dispute/ Harmony	Dispute/ Tension	Results
Liu, 2018	<120,000 Yuan per year<br>120,000–240,000 Yuan per year<br>>240,000 Yuan per year	15,784<br>2969<br>793	18,771<br>4600<br>1169	Significant difference (χ^2 test-p<0.05) 120,000–240,000 Yuan vs. <120,000 Yuan (OR = 1.186, 95%CI: 1.079–1.304)
Shi, 2017	≤3000 Yuan per month<br>3000–5000 Yuan per month<br>5000–10 000 Yuan per month<br>>10 000 Yuan per month	2970<br>2176<br>311<br>11	5693<br>4186<br>603<br>20	No significant difference (χ^2 test-p>0.05)

Yuan, 2017	<2000 Yuan per month<br>2000–3999 Yuan per month<br>4000-6000 Yuan per month<br>>6000 Yuan per month	303<br>487<br>132<br>20	570<br>917<br>202<br>19	No significant difference (χ^2 test = 7.454, p = 0.059)

Table S15. A10-Income Satisfaction

Author	Group	No Dispute/ Harmony	Dispute/ Tension	Results
Zhao, 2017	dissatisfied<br>satisfied	69<br>45	187<br>219	Significant difference (χ^2 test-p<0.05)

Table S16. A11-Working Time Per Day

Author	Group	No Dispute/ Harmony	Dispute/ Tension	Results
Gao, 2015	<8 hours<br>8-10 hours<br>10–12 hours<br>>12 hours	3<br>21<br>3<br>1	11<br>25<br>51<br>45	Significant difference (χ^2 test = 38.221, p = 0.000)
Shi, 2017	0–2 hours<br>2–4 hours<br>4–6 hours<br>6–8 hours<br>>8 hours	97<br>149<br>159<br>2492<br>2571	119<br>223<br>218<br>4308<br>5634	Significant difference (p< 0.05)

Table S17. A12-Marital Status

Author	Group	No Dispute/ Harmony	Dispute/ Tension	Results
Shi, 2017	Married<br>Unmarried<br>Divorced or widowed	3274<br>2111<br>83	6697<br>3646<br>159	Significant difference in tertiary hospitals (χ^2 test = 27.8, p<0.001)
Yuan, 2017	Married<br>Unmarried	246<br>698	418<br>1304	No significant difference (χ^2 test = 1.039, p = 0.3081)

Table S18. A13-Employment Form

Author	Group	No Dispute/ Harmony	Dispute/ Tension	Results
Shi, 2017	Regular staff<br>Temporary employee	2913<br>2555	5503<br>4999	No significant difference (χ^2 test-p> 0.05)
Yuan, 2017	Officially enrolled employee<br>Contract employee<br>Temporary employee<br>Other	494<br>379<br>35<br>30	845<br>782<br>67<br>28	Significant difference (χ^2 test = 11.967, p< 0.008)

Table S19. A14-Administrative Position

Author	Group	No Dispute/ Harmony	Dispute/ Tension	Results
Liu, 2018	Yes<br>No	2,290<br>17,256	2670<br>21,870	Significant difference (χ^2 test-p< 0.05) Yes vs. No (OR = 1.326, 95%CI:1.195–1.471)

Table S20. A15-Whether Disputed with Patient

Author	Group	No Dispute/ Harmony	Dispute/ Tension	Results
Zhao, 2017	Yes<br>No	85<br>24	369<br>42	Significant difference (χ^2 test-p< 0.05)
Gao, 2015	Yes<br>No	23<br>5	97<br>35	No significant difference (χ^2 test = 0.924, p = 0.337)

Table S21. A16-Workload

Author	Group	No Dispute/ Harmony	Dispute/ Tension	Results
Zhao, 2017	Heavy<br>Light	81<br>21	367<br>51	Significant difference (χ^2 test-p< 0.05)
Gao, 2015	Heavy<br>Light	15<br>13	107<br>25	Significant difference (χ^2 test = 9.629, p = 0.002)

Table S22. A17-Medical Ethics

Author	Group	No Dispute/ Harmony	Dispute/ Tension	Results
Zhao, 2017	Good<br>Poor	72<br>31	140<br>277	Significant difference (χ^2 test-p< 0.05)

Table S23. A18-Whether Medical Disputes Interfere with Work

Author	Group	No Dispute/ Harmony	Dispute/ Tension	Results
Zhao, 2017	Yes<br>No	88<br>35	326<br>71	Significant difference (χ^2 test-p< 0.05)

Table S24. A19-Career Satisfaction

Author	Group	No Dispute/ Harmony	Dispute/ Tension	Results

Wang, 2015	>80<br>60-80<br><60	45<br>238<br>129	33<br>112<br>61	No significant difference (χ^2 test = 3.236, p = 0.198)

Table S25. A20-Ability to Handle Dispute

Author	Group	No Dispute/ Harmony	Dispute/ Tension	Results
Wang, 2015	Good<br>Poor	367<br>45	173<br>33	No significant difference (χ^2 test = 3.235, p = 0.072)

Table S26. A21-Whether Worry about Encountering Dispute

Author	Group	No Dispute/ Harmony	Dispute/ Tension	Results
Gao, 2015	Yes<br>No	18<br>10	132<br>0	Significant difference (χ^2 test = 50.286, p = 0.000)

Table S27. A22-Daily Average Rate of Outpatient Visits

Author	Group	No Dispute/ Harmony	Dispute/ Tension	Results
Gao, 2015	3–5<br>5–7<br>7–9<br>≥10	1<br>7<br>1<br>19	14<br>7<br>10<br>101	Significant difference (χ^2 test = 12.231, p = 0.007)

Table S28. A23-Medical Liability Insurance

Author	Group	No Dispute/ Harmony	Dispute/ Tension	Results
Liu, 2018	Yes<br>No<br>Unclear	12,994<br>1742<br>4810	11,118<br>3450<br>9972	Significant difference (χ^2 test-p< 0.05) Yes vs. No (OR = 2.193, 95%CI: 2.066–2.327)

Table S29. A24-Time Spent in Direct Contact with the Patient

Author	Group	No Dispute/ Harmony	Dispute/ Tension	Results
Shi, 2017	0–2 hours<br>2–4 hours<br>4–6 hours<br>6–8 hours	162<br>217<br>518<br>4571	124<br>271<br>804<br>9303	Significant difference (χ^2 test-p< 0.001)

PERSPECTIVE OF PATIENT

Table S30. B1-Gender

Author	Group	No Dispute/ Harmony	Dispute/ Tension	Results
Wang, 2015	Male<br>Female	196<br>216	109<br>97	No significant difference (χ^2 test = 1.566, p = 0.211)
Deng, 2010	Male<br>Female	37<br>23	44<br>36	No significant difference (χ^2 test = 1.238, p = 0.539)
Zhao, 2018	Male<br>Female	6701<br>13,651	2789<br>5964	No significant difference (χ^2 test = 3.140, p = 0.080)

Table S31. B2-Age

Author	Group	No Dispute/ Harmony	Dispute/ Tension	Results
Wang, 2015	<18<br>18–60<br>>60	38<br>246<br>128	21<br>110<br>71	No significant difference (χ^2 test = 1.536, p = 0.464)
Deng, 2010	<20<br>20–39<br>40–59<br>>60	3<br>40<br>11<br>6	2<br>43<br>22<br>13	No significant difference (χ^2 test = 10.882, p = 0.094)
Zhao, 2018	<35<br>35–65<br>>65	10,529<br>8159<br>1664	4569<br>3492<br>692	No significant difference (χ^2 test = 0.86, p = 0.65)
Yang, 2014	≤19<br>20–39<br>40–59<br>≥60	92<br>676<br>448<br>120	21<br>131<br>42<br>9	Significant difference (χ^2 test = 22.875, p = 0.000)

Table S32. B3-Registered Residence

Author	Group	No Dispute/ Harmony	Dispute/ Tension	Results
Wang, 2015	Urban<br>Rural	153<br>259	62<br>144	No significant difference (χ^2 test = 2.999, p = 0.083)
Deng, 2010	Urban<br>Rural	13<br>47	45<br>35	Significant difference (χ^2 test = 17.096, p = 0.000)
Zhao, 2018	Urban	14,538	6085	Significant difference

	Rural	5,814	2,668	(χ^2 test= 10.860, p<0.01)

Table S33. B4-Education Level

Author	Group	No Dispute/ Harmony	Dispute/ Tension	Results
Wang, 2015	Primary school or below	165	103	Significant difference (χ^2 test = 7.355, p = 0.025)
	Junior middle school	131	63	
	High school or above	116	40	
Deng, 2010	Primary school	8	8	Significant difference (χ^2 test = 30.254, p =0.000)
	Junior middle school	31	15	
	High school	14	30	
	Junior college	5	10	
	Undergraduate or above	2	17	
Zhao, 2018	Junior high school or below	4194	1434	Significant difference (χ^2 test = 132.13, p<0.01)
	Senior high school	5351	2050	
	Undergraduate or above	10,807	5269	
Liu, 2010	Junior college or below	956	258	Significant difference (χ^2 test = 7.725, p = 0.021)
	Undergraduate	224	43	
	Master or above	31	2	
Yang, 2014	Illiteracy	49	5	Significant difference (χ^2 test = 19.001, p = 0.004)
	Primary school	115	17	
	Junior middle school	492	60	
	Polytechnic school	158	22	
	High school	271	39	
	Junior college	136	24	
	Undergraduate or above	115	36	

Table S34. B5-Occupation

Author	Group	No Dispute/ Harmony	Dispute/ Tension	Results
Wang, 2015	No regular occupation	229	137	Significant difference (χ^2 test = 6.838, p = 0.032)
	Student	45	18	
	Have regular occupation	138	51	

Table S35. B6-Medical Insurance

Author	Group	No Dispute/ Harmony	Dispute/ Tension	Results
Wang, 2015	None	34	26	Significant difference (χ^2 test = 7.100, p = 0.029)
	New rural cooperative medical system	240	98	
	Other medical insurance	138	82	
Deng, 2010	Medical insurance for Urban residents	2,635	1242	Significant difference (χ^2 test = 35.100, p< 0.01)
	Medical insurance for urban employees	2,482	884	
	New rural cooperative medical system	6188	2743	
	Commercial medical insurance	8716	3717	
	None	331	167	
Liu, 2010	No	470	153	Significant difference

	Yes	741	150	(χ^2 test-p< 0.05)
Yang, 2014	Medical insurance for urban employees<br>Medical insurance for Urban residents<br>New rural cooperative medical system<br>Other medical insurance	285<br>192<br>821<br>38	39<br>31<br>118<br>15	Significant difference (χ^2 test = 11.364, p = 0.01)

Table S36. B7-Medical Expenses

Author	Group	No Dispute/ Harmony	Dispute/ Tension	Results
Zhang, 2011	Very satisfied<br>Satisfied<br>General<br>Dissatisfied<br>Very dissatisfied	28<br>152<br>77<br>6<br>1	17<br>87<br>98<br>31<br>1	Significant difference (χ^2 test = 38.110, p = 0.000)
Wang, 2015	<5000 Yuan<br>5000–10000 Yuan<br>10000–20000 Yuan<br>>20000 Yuan	28<br>81<br>214<br>79	15<br>38<br>90<br>63	Significant difference (χ^2 test = 9.671, p = 0.022)

Table S37. B8-Household Income

Author	Group	No Dispute/ Harmony	Dispute/ Tension	Results
Deng, 2010	<800 Yuan per month<br>800–2000 Yuan per month<br>2000–3000 Yuan per month<br>>3000 Yuan per month	34<br>20<br>4<br>2	23<br>28<br>16<br>13	Significant difference (χ^2 test = 17.037, p = 0.009)
Zhao, 2018	<60000 Yuan per year<br>60000–120000 Yuan per year<br>>120000 Yuan per year	12,639<br>4586<br>3127	5109<br>2121<br>1523	Significant difference (χ^2 test = 37.52, p<0.01) >120000 Yuan vs. <60000 Yuan (OR = 0.83, 95%CI: 0.75–0.91)

Table S38. B9-Whether Have a Familiar Doctor

Author	Group	No Dispute/ Harmony	Dispute/ Tension	Results
Zhao, 2018	Yes<br>No	6777<br>13,575	2351<br>6402	Significant difference (χ^2 test = 117.91, p<0.01) Yes vs. No (OR = 0.88, 95%CI:0.82–0.95)
Zhang, 2011	Yes<br>No	153<br>111	99<br>135	Significant difference (χ^2 test = 12.15, p = 0.000)

Table S39. B10-Hospital Type

Author	Group	No Dispute/ Harmony	Dispute/ Tension	Results

Zhao, 2018	The general hospital	8551	3432	Significant difference (χ^2 test = 77.53, p<0.01)
	The traditional Chinese medicine hospital	4856	1870	
	The women and children's hospital	4776	2408	
	Other specialized hospitals	2169	1043	

Table S40. B11-Region

Author	Group	No Dispute/ Harmony	Dispute/ Tension	Results
Zhao, 2018	The eastern region	8970	3351	Significant difference (χ^2 test = 108, p<0.01) The western region vs. The eastern region (OR = 0.81, 95%CI:0.75–0.87)
	The central region	4985	2154	
	The western region	6397	3248	

Table S41. B12-Department

Author	Group	No Dispute/ Harmony	Dispute/ Tension	Results
Zhao, 2018	Internal medicine	5711	2210	Significant difference (χ^2 test = 100.49, p<0.01) Paediatrics vs. Internal medicine (OR = 0.82, 95%CI:0.71–0.95)
	Surgery	2143	794	
	Gynaecology and obstetrics	4416	2150	
	Paediatrics	1673	860	
	Traditional Chinese Medicine	1300	423	
	Other	5109	2316	

Table S42. B13-Whether First Visit

Author	Group	No Dispute/ Harmony	Dispute/ Tension	Results
Zhao, 2018	Yes	8979	4122	Significant difference (χ^2 test = 21.87, p<0.01) Yes vs. No (OR = 1.09, 95%CI:1.01–1.17)
	No	11,373	4631	

Table S43. B14-Registration

Author	Group	No Dispute/ Harmony	Dispute/ Tension	Results
Zhao, 2018	Through making an appointment	11,601	4987	No significant difference (χ^2 test = 0.000, p = 0.97)
	Through Service windows	8751	3766	

Table S44. B15-Sources of Patients

Author	Group	No Dispute/ Harmony	Dispute/ Tension	Results
Zhao, 2018	Local population with registration	12,746	5396	Significant difference

	Local population without registration	3421	1601	(χ^2 test = 9.49, p<0.01)
	Non-local population	4185	1756	

Table S45. B16-Whether Have Family Doctors

Author	Group	No Dispute/ Harmony	Dispute/ Tension	Results
Zhao, 2018	Yes<br>No	1218<br>19,134	332<br>8421	Significant difference (χ^2 test = 117.91, p<0.01) Yes vs. No (OR = 0.72, 95%CI: 0.61–0.84)

Table S46. B17-Referral

Author	Group	No Dispute/ Harmony	Dispute/ Tension	Results
Zhao, 2018	Yes<br>No	19,523<br>829	8399<br>354	No significant difference (χ^2 test = 0.01, p = 0.91)

Table S47. B18-Operation

Author	Group	No Dispute/ Harmony	Dispute/ Tension	Results
Wang, 2015	Yes<br>No	242<br>170	128<br>78	No significant difference (χ^2 test = 0.66, p = 0.417)

PERSPECTIVE OF THERAPEUTIC INTERACTION

Table S48. C1-Health Worker-Patient Trust

Author	Group	No Dispute/ Harmony	Dispute/ Tension	Results
Zhao, 2017	Trust<br>General<br>Distrust	72<br>24<br>7	211<br>157<br>59	Significant difference (χ^2 test-p<0.05)
Zhang, 2011	0–4<br>5–6<br>7–8<br>9–10	8<br>35<br>121<br>100	9<br>61<br>121<br>43	Significant difference (χ^2 test = 28.116, p = 0.000)
Yang, 2014	Very trust<br>Trust<br>Distrust<br>Very Distrust	303<br>960<br>68<br>5	11<br>117<br>62<br>13	Significant difference (χ^2 test= 220.752, P=0.000)

Table S49. C2-Service Attitude

Author	Group	No Dispute/ Harmony	Dispute/ Tension	Results
Zhang, 2011	Very satisfied	45	25	Significant difference (χ^2 test = 19.293, *p* = 0.001)
	Satisfied	174	134	
	General	39	69	
	Dissatisfied	5	4	
	Very dissatisfied	1	3	
Wang, 2015	Good	282	112	Significant difference (χ^2 test = 11.778, *p* = 0.001)
	Poor	130	94	

Table S50. C3-Service Quality and Level

Author	Group	No Dispute/ Harmony	Dispute/ Tension	Results
Zhang, 2011	Very satisfied	45	23	Significant difference (χ^2 test = 14.358, *p* = 0.006)
	Satisfied	182	150	
	General	34	56	
	Dissatisfied	2	3	
	Very dissatisfied	1	2	

Table S51. C4-Treatment Effect

Author	Group	No Dispute/ Harmony	Dispute/ Tension	Results
Zhang, 2011	Very satisfied	39	22	Significant difference (χ^2 test = 16.632, *p* = 0.002)
	Satisfied	185	147	
	General	36	58	
	Dissatisfied	4	3	
	Very dissatisfied	0	4	

Table S52. C5-Whether Health Worker Receives Kickbacks on Medications or Medical Devices

Author	Group	No Dispute/ Harmony	Dispute/ Tension	Results
Zhang, 2011	Yes	148	153	Significant difference (χ^2 test = 4.519, *p* = 0.034)
	No	116	81	

Table S53. C6-Whether Adequate Medical Information is Shared

Author	Group	No Dispute/ Harmony	Dispute/ Tension	Results
Zhang, 2011	Yes	249	208	Significant difference (χ^2 test = 4.84, *p* = 0.028)
	No	15	26	

Table S54. C7-Health Worker-Patient Communication

Author	Group	No Dispute/ Harmony	Dispute/ Tension	Results
Yang, 2014 [75]	1 (Good)	243	8	Significant difference (χ^2 test = 238.5, p = 0.000)
	2	998	108	
	3	88	69	
	4 (Poor)	7	18	

Table S55. C8-Whether Patient Bribes or Entertains Doctors

Author	Group	No Dispute/ Harmony	Dispute/ Tension	Results
Zhang, 2011 [74]	Yes	124	137	Significant difference (χ^2 test = 6.666, p = 0.01)
	No	140	97	
